# Clinical characteristics of chemsex users attended in a ngo in madrid

**DOI:** 10.1192/j.eurpsy.2023.1339

**Published:** 2023-07-19

**Authors:** J. Curto Ramos, L. Ibarguchi, P. Barrio, A. García, M. A. Morillas Romerosa, P. Herrero, H. Dolengevich Segal

**Affiliations:** 1 Department of Psychiatry, Clinical Psychology and Mental Health, La Paz University Hospital, Madrid, Spain, Hospital la Paz; 2 NGO Apoyo Positivo; 3 Hospital la Paz, Madrid; 4 Department of Psychiatry, Clinical Psychology and Mental Health, Hospital del Henares, Coslada, Spain

## Abstract

**Introduction:**

The intentional use of drugs before or during sexual intercourse (chemsex) is a phenomenon of special importance in the MSM (men who have sex with men) population due to its impact on mental, physical and sexual health.

**Objectives:**

The objective of this study was to describe the sociodemographic and medical characteristics, psychoactive substances use of a sample of users with sexualized drug use (chemsex) attended by the non-govenrmental organization Apoyo Positivo in the program “Sex, Drugs and You”.

**Methods:**

A cross-sectional descriptive analysis of a sample of users attended by the non-govenrmental organization Apoyo Positivo in the program “Sex, Drugs and You” was performed.

**Results:**

230 participants were included. Most common drugs used during sexual intercourse were: mephedrone, cocaine, poppers, GHB and methamphetamine. The frequencies of substances consumed during sex were: mephedrone (95%), methamphetamine (80%), GHB (92.2%), ketamine (52%), poppers (alkyl nitrites) (95%), cocaine (89 .7%), speed (amphetamine sulfate) (49.6%) and drugs for erectile dysfunction (86%). 61.3% reported having practiced slamsex intravenous substance use at some time in their life, being a habitual practice at the time of collecting information for 50.7%. The most frequent genitally transmitted infections were: syphilis, chlamydia and gonorrhea. Users reported having been diagnosed with the following genitally transmitted infections: hepatitis B virus (7.4%), hepatitis A virus (18.6%), syphilis (69.6%), human papillomavirus (16 %), herpes (9.4%), chlamydia (43%), gonorrhea (60.5%) and candidiasis (9.7%).

**Conclusions:**

Slamsex and STIs are usually reported in our sample. Interventions for chemsex users must include a colaborative model which includes professionals from different areas, including internists and emergency physicians, psychiatrists, psychologists, nurses, social workers and sexologists.

**Disclosure of Interest:**

None Declared

